# Research on Volatile Allergenic Substances in Chinese Lacquer: An Integrated Analysis of Their Composition, Detection, Mechanisms, and Prevention

**DOI:** 10.3390/polym17131722

**Published:** 2025-06-20

**Authors:** Yao Wang, Jiangyan Hou, Tianyi Wang, Xinhao Feng, Xinyou Liu

**Affiliations:** 1 College of Furnishing and Industrial Design, Nanjing Forestry University, Nanjing 210037, China; 2381132433@njfu.edu.cn (Y.W.); houjiang@njfu.edu.cn (J.H.); 13861210436@njfu.edu.cn (T.W.); liu.xinyou@njfu.edu.cn (X.L.); 2 Co-Innovation Center of Efficient Processing and Utilization of Forest Resources, Nanjing Forestry University, Nanjing 210037, China

**Keywords:** Chinese lacquer, volatile organic compounds, allergy mechanism, gas chromatography-mass spectrometry (GC/MS)

## Abstract

As a natural polymerized material, Chinese lacquer has numerous applications, although its processing is associated with volatile organic compounds (VOCs), which will cause a health risk. This paper was mainly focused on the detection of volatiles in the Chinese lacquer and its possible allergy mechanisms based on the properties of the lacquer, such as the main components, chemical properties, and allergy mechanisms of the unit phenols, aldehydes, and ketones and terpenes in the volatiles. Based on the detection technology (such as GC/MS) and allergy mechanism, a variety of prevention and control strategies are proposed, including the use of cyclodextrin–chitosan embedding technology to reduce the antigenicity of lacquer phenols and the directional modification of the active site of laccase to inhibit the generation of quinone toxicity products, as well as the research and development of antioxidant protective equipment for different volatiles, the installation of ventilation and purification devices, and the addition of antioxidants. They are all aimed at providing scientific evidence and practical guidance for the safe use of lacquer, the health protection of the practitioners, and the sustainable development of the related industries.

## 1. Introduction

Chinese lacquer, a natural polymer material, forms highly durable coatings with exceptional aesthetic and functional properties [1,2,3,4,5]. Despite its significance in cultural heritage preservation, lacquer processing releases volatile organic compounds (VOCs)—including terpenoids, aldehydes, and ketones—that pose allergy, respiratory, and neurological risks [6].

The conventional detection methodologies for volatile substances primarily encompass gas chromatography (GC), high-performance liquid chromatography (HPLC), and mass spectrometry (MS), with combined configurations such as GC/MS and LC/MS being conventionally employed due to their acknowledged reliability in compound separation and structural elucidation [7,8]. Gas chromatography (GC) stands as one of the most prevalent analytical techniques, excelling in separating and quantifying volatile constituents within Chinese lacquer [9].

The chemical profiles of volatile substances in Chinese lacquer are defined by their molecular architecture, polarity, volatility, and reactivity. These constituents predominantly comprise organic compounds such as alcohols, ketones, aldehydes, and aromatic hydrocarbons.

**Figure 1:**
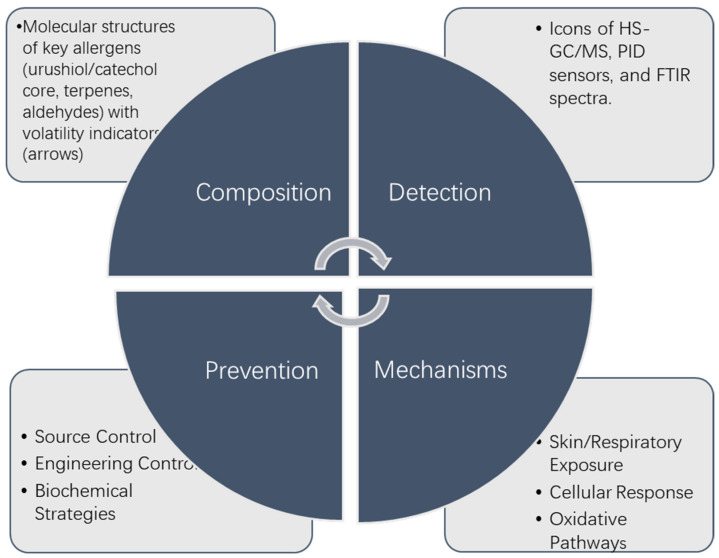
Research structure.

This study adopted an interdisciplinary approach to investigate the detection technologies, allergy mechanisms, and prevention strategies for volatile substances in Chinese lacquer. By analyzing the chemical properties of volatile substances and their impacts on human health and the environment, the research aim was to elucidate their potential ecological risks. Finally, this paper proposes source control measures for volatile substances in Chinese lacquer and safety management strategies during its application, and discusses supportive policies and regulations, providing scientific foundations and solutions for the safe utilization of Chinese lacquer. The structure of the study is shown in Figure 1.

## 2. Main Components and Characteristics of Chinese Lacquer

### 2.1. Composition and Structure of Chinese Lacquer

#### 2.1.1. Composition of Lacquer

Lacquer sap is separated into functional components through a sequential solvent extraction process, whereby an acetone treatment first isolates soluble lipids (60–70%, primarily urushiol—the film-forming agent) and water (20–30%), leaving acetone-insoluble “acetone powder” [10]. This powder is further fractionated with water, yielding water-soluble plant gums (4–10%, with the polysaccharides providing viscosity) and enzymes (1.5–2%, including laccase or peroxidase for urushiol polymerization and stellacyanin for redox regulation), alongside water-insoluble glycoprotein (3–5%, enhancing the film integrity). This structured separation reveals how lacquer’s self-curing mechanism relies on urushiol–enzyme coexistence, where exposure to oxygen or humidity triggers polymerization into a durable, waterproof coating.

#### 2.1.2. Chemical Structure of Lacquer

A comparative study [6] analyzed the major lipoid constituents of three oriental lacquer tree saps—*Rhus vernicifera*, *Rhus succedanea*, and *Melanorrhoea usitata*. It was found that the main lipid-like components of the three-lacquer tree sap have a catechol structure (benzene ring + 1,2-position hydroxyl group) as the core skeleton, and their sensitizing activity and film-forming properties are directly related to this structure, although there are obvious differences in the types of side chains (as shown in Table 1 below).

### 2.2. Properties of Lacquer

#### 2.2.1. Morphological Characteristics of Lacquer

The morphological characteristics of lacquers are key aspects that affect their overall performance and application. Large lacquers often exhibit complex structures, and their film-forming properties are key to creating a smooth and durable surface. When lacquer exudates from lacquer trees dry, they produce a tough, lustrous film, and they have been used as natural coatings [11].

**Figure 2:**
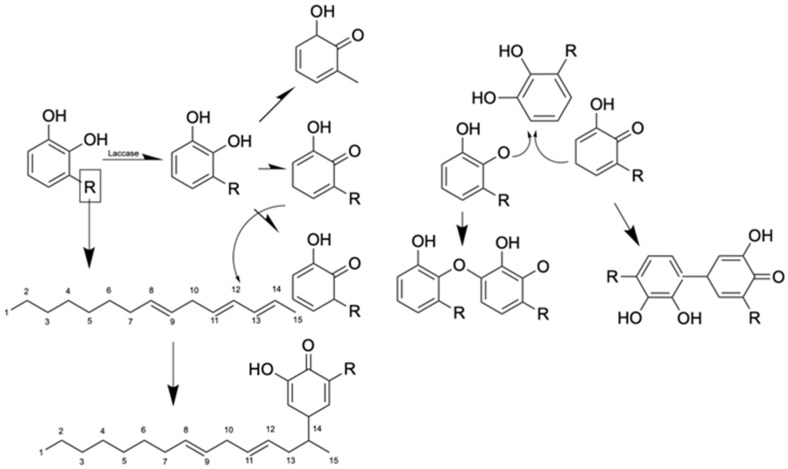
Mechanism of oxidation and polymerization of urushiol.

Lacquer sap forms its signature durable film through enzymatic polymerization, whereby laccase and peroxidase catalyze the oxidation of urushiol lipids (60–70%), triggering crosslinking into a hydrophobic, insoluble polymer network upon exposure to oxygen and humidity. Concurrently, toxicity arises as urushiol acts as a potent contact allergen, penetrating the skin and binding to proteins to form hapten–carrier complexes that provoke immune-mediated allergic contact dermatitis in sensitized individuals, as shown in the reaction in Figure 2.

#### 2.2.2. Gloss Properties

The gloss properties are important physical properties of lacquers, directly affecting their visual appeal and market value. The gloss level of lacquers ranges from matte to high-gloss, depending on their formulation and coating technology. The glossiness of a lacquer film is an important index to evaluate its surface quality. Experimental data [12] indicate that the initial gloss of single-layer unmodified lacquer films (blank lacquer) measures 68 (60° angle), while organo-silane-modified hybrid films achieve a value of 77. Double-layer coatings show further increases, with unmodified films at 88 and modified films reaching 93. A study [13] on car coatings revealed gloss levels above 70 after 1000 days of use, although significant declines occurred during rainy and summer seasons. UV irradiation experiments [14] demonstrated that prolonged exposure induces annular cracks and white spots on film surfaces, correlating with increased oxidizing groups (e.g., C-O, C=O) in XPS analyses, which suggests polymer network degradation as the primary cause of gloss loss.

#### 2.2.3. Mechanical Properties

The mechanical properties of lacquers are critical in determining their suitability for a variety of applications. The key mechanical properties include the hardness, flexibility, adhesion, and impact resistance.

Hardness is an important factor affecting the durability of large lacquer films, and coatings with higher hardness are better able to resist scratches and abrasion. A previous study employed two methods—ASTM standard pencil hardness testing and quantitative nanoindentation measurements. The results showed that Korean lacquer exhibited the highest hardness, reaching 3H after 3 days of drying and increasing to 4H after 21 days. Vietnamese lacquer, which was initially softer at 2B hardness, also achieved 4H after 21 days of drying [15].

The impact resistance measures the ability of a large paint to withstand physical impact without cracking or peeling. These mechanical properties are influenced by the formulation of the lacquer, including the choices of resin, solvent, and additives, as well as the curing conditions. A study [16] revealed that laccol-based lacquer films exhibit crosslinking reactions over three times slower than urushiol-based ones, with the hardening time extending from 24 to 72 h. This slower crosslinking leads to looser internal structures, as confirmed by FT-IR data showing the slower disappearance of carbon–carbon double bonds and lower crosslinking density, potentially reducing the film impact strength.

#### 2.2.4. Corrosion Resistance

Chinese lacquer exhibits remarkable corrosion resistance. Multimodal analytical techniques—including SEM-EDS, FT-IR, and Py-GC/MS with TMAH derivatization—were employed to analyze the organic and inorganic components of ancient lacquerware [17]. It was revealed that the exceptional corrosion resistance of ancient Chinese lacquer originates from its multicomponent synergistic protection system, whereby the oxidative polymerization of urushiol forms a dense three-dimensional hydrophobic network that effectively blocks moisture penetration.

#### 2.2.5. Weather Resistance

Weather resistance represents another critical chemical property of lacquer. The lacquer formulation typically incorporates UV stabilizers and antioxidants, which help mitigate film degradation caused by solar exposure. The lacquer film exhibits remarkable resistance to ultraviolet (UV) light. Accelerated aging tests demonstrate that after 1000 h of irradiation in the 315–400 nm wavelength range, only micro-cracks (SEM reveals crack widths < 1 μm) appear on the film surface. This UV resistance surpasses that of synthetic polyurethane coatings (crack widths > 5 μm under identical conditions) [18].

#### 2.2.6. Insulation Properties

Lacquer also exhibits remarkable insulating properties, contributing to its utility in diverse applications. The chemical structure of lacquer enables it to function as an electrical insulator, making it a valuable coating material for electrical components and wiring. This insulating capability is particularly crucial for preventing short circuits and ensuring the safety of electrical systems. In modern Japan, lacquer is employed as both an insulating and anti-corrosion material [19]. Native lacquer exhibits a biphasic microstructure, whereby polysaccharide regions form porous, networked frameworks at the micrometer scale, enhancing the material flexibility and stress dissipation, while urushiol domains self-assemble into dense, homogeneous nanoscale matrices through crosslinking. This structure creates synergistic properties; the polysaccharide phase absorbs mechanical strain, whereas the continuous urushiol matrix provides exceptional barrier resistance against moisture and chemicals, electrical insulation, and mechanical robustness.

#### 2.2.7. Allergy

Allergy to Chinese lacquer constitutes a central issue in its toxicity research. Urushiol, as the primary sensitized component, exhibits structure–activity relationships where its catechol core is combined with structural features of the unsaturated long side chain (particularly the C15–C17 side chain containing 1–3 double bonds), which directly determines its allergy potency [20]. The allergy pathways mainly include three types—direct contact allergy (skin contact with urushiol), indirect contact allergy (transfer of contaminants to sensitive areas), and airborne allergy (volatile substances penetrating through the respiratory tract or mucous membranes).

The allergy potential of lacquer is closely related to its chemical state. Environmental factors also play a crucial role, whereby high temperature and humidity levels accelerate the oxidation of urushiol into highly reactive quinones, while deteriorated lacquer may release secondary sensitized metabolites [21,22].

Lacquer allergy typically refers to allergic contact dermatitis (ACD) induced by the lipid components present in raw lacquer sap. From an immunological perspective, these lacquer lipid constituents function as haptens [22]. When urushiol contacts the skin, its unique chemical structure binds to skin cells to form. The severity of the allergic reaction varies among individuals, and long-term exposure to raw lacquer increases the likelihood of severe symptoms in practitioners.

Urushiol (a hapten) penetrates the skin and oxidizes into electrophilic *o*-quinones, which covalently bind to nucleophilic groups (e.g., -NH_2_) on membrane proteins, forming complete antigens (neoantigens). These complexes are processed by dendritic cells, activating T-cells and establishing immune memory. Upon re-exposure, memory T-cells recognize the antigens, triggering cytokine release and inflammation that manifests as allergic contact dermatitis flare-ups—characterized by delayed hypersensitivity reactions days after exposure.

It is confirmed by immunological research [23] that urushiol-derived ortho-quinones are highly susceptible to nucleophilic attacks at the 4-, 5-, and 6-positions of the catechol ring by protein residues, forming complete antigens. The mechanism of allergic contact dermatitis has been significantly advanced through mechanistic studies [24,25].

Multiple innovative strategies for lacquer allergy prevention and control have been proposed in the recent research. Heat treatment has been demonstrated [22] to destroy urushiol’s antigenic epitopes, significantly reducing lacquerware allergy, while urushiol encapsulation technology (e.g., cyclodextrin modification) has been proposed [20] to block its binding to skin proteins. These insights provide critical theoretical foundations for occupational health protection and safe lacquer utilization.

#### 2.2.8. Antimicrobial Properties

Urushiol, the primary constituent (60–70% by weight) of Chinese lacquer sap, exhibits notable antibacterial and antioxidant properties. It was demonstrated [26] that a poly(urethane-urea) (PUU) film with 22.2 wt.% urushiol (UR-3) showed antibacterial activity indices (R-values) of 2.3 (99.4% reduction) against Staphylococcus aureus (ATCC 6538) and 1.6 (97.7% eradication) against Escherichia coli (ATCC 25922). Urushiol’s superior antibacterial efficacy against Helicobacter pylori (clinical strain HP26695) with minimum inhibitory concentrations (MIC) below 8 μg/mL has been further confirmed [27].

#### 2.2.9. Drying Mechanisms and Construction Performance

The drying mechanism of Chinese lacquer is a critical factor influencing its performance in practical applications. Chinese lacquer typically dries through a combination of solvent evaporation and chemical curing processes. After applying raw lacquer, the coated object is placed in a drying chamber maintained at 70% relative humidity (RH) and 20–25 °C, where the lacquer undergoes gradual oxidation. Approximately 5–8 h later, the surface reaches a tack-free stage (no fingerprint residue upon gentle touch). However, the coating is not fully cured at this stage; applying slight pressure will still leave marks. The oxidation reaction continues until the coating achieves complete curing, characterized by full hardness and chemical stability [28]. The application performance of lacquer is reflected in its adhesion to various substrates, flexibility, and resistance to cracking and peeling. These properties are crucial for ensuring long-lasting surface effects in both indoor and outdoor applications, as exposure to moisture and temperature fluctuations can significantly affect the durability in these environments. Its superior properties, including its high durability, strength, water resistance, and antibacterial levels, have been proven by the fact that traditional lacquerware retains its shape and appearance without damage even after 2000 years [29].

#### 2.2.10. Decorative Applications

As recorded in Yang Ming’s Preface to Xiushi Lu, “Whether employed in ceremonial vessels, weaponry, architectural structures, or mortuary artifacts, lacquerware achieves its distinction through dual excellence: material durability for structural integrity, and decorative radiance for aesthetic transcendence” [30]. Its capacity to produce smooth, uniform coatings renders it a preferred choice for surface treatments in furniture, cabinetry, and automotive applications.

### 2.3. Chinese Lacquer Volatile Compounds

Chinese lacquer releases various VOCs during use and drying processes, which not only affect its odor but may also pose potential health risks. It has been shown that water-based, lacquer-coated veneer particleboards emit multiple VOCs during use, including aldehydes, ketones, and terpenes. Aldehyde and ketone compounds exhibit strong bioactivity, readily irritating respiratory mucosa and triggering inflammatory responses, while terpenes may exacerbate allergic reactions via immune system modulation [26].

Capillary chromatography column separation technology was employed to analyze volatile compounds released from raw lacquer at 40 °C [31]. The primary components were identified as bromoethane, acrolein, acrylic acid, formic acid, acetic acid, and butanol. A total of 29 peaks were detected in the total ion chromatogram (TIC) data of raw lacquer, with a mass spectrometric analysis confirming components including alcohols, esters, organic acids, monoterpenes, and sesquiterpenes [32]. The analytical results are summarized in Table 2, and the potential allergy mechanisms of these substances will be discussed in subsequent sections.

#### 2.3.1. Unit Phenols

Although phenolic compounds exhibit extremely low volatility, trace amounts can still persist in raw lacquer emissions under summer conditions of high humidity and poor ventilation, attributable to the presence of monomeric phenols in raw lacquer [33,34].

The volatility of lacquer phenols is very low, so the chance of respiratory and oral mucous membrane inhalation is very small. Unitary phenols in raw lacquer [33] are more volatile than catechol, and only 0.001 mg of raw lacquer entering the body [35] can lead to skin rashes in highly allergic individuals.

Monophenols are not a core sensitizing component in lacquer systems, and their presence is scarce and their biological activity is limited.

#### 2.3.2. Aldehydes and Ketones

Aldehydes and ketones, as toxic pollutants, are extremely harmful to the environment. According to statistics, the number of current standards in this area reaches as many as 14 kinds, whose molecular structures contain an aldehyde group (-CHO) or carbonyl group (C=O), respectively, and can be classified into two major categories of aldehydes and ketones according to their chemical characteristics [36]. The specific classification is shown in the following Table 3.

Aldehydes and ketones include aldehydes and ketones. Aldehydes are substances that exist as compounds with a hydrocarbon group and an aldehyde group linked in the molecular structure. Ketones are substances in the form of compounds with a hydroxyl group and two hydrocarbon groups linked in the molecular structure [37].

For aldehydes and ketones, their physio-chemical properties are closely related to their molecular structure. Specific physical properties are shown in Table 4.

#### 2.3.3. Terpenes

**Figure 3:**
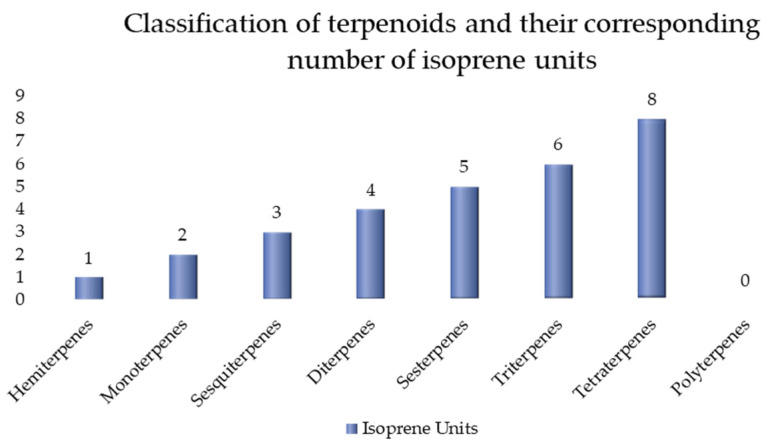
Classification of terpenoids and their corresponding numbers of isoprene units.

Most terpenes have isoprene (2-methyl-1,4-butadiene) as a common carbon skeleton building block. Wallach discovered this structural feature as early as 1887 and pointed out that most terpenes are formed by the condensation of isoprene units in a “head-to-tail” manner, a rule known as the “isoprene rule”. Based on this rule, terpenes can be classified according to the number of isoprene units they contain [38], as shown in Figure 3.

Terpenoid classification by isoprene units directly governs key properties: Volatility decreases with size (e.g., monoterpenes [C_10_] evaporate readily; triterpenes [C_30_] are non-volatile). Reactivity increases with unsaturation or functional groups; sesquiterpenes (C_15_) and diterpenes (C_20_) often bear epoxides or aldehydes enabling electrophilic binding (allergenicity). Health effects correlate, whereby smaller terpenes (C_5_–C_10_) act as irritants or vapor toxins; larger (C_30_+) structures exhibit bioactivity (e.g., triterpene anti-inflammatories) but may accumulate in tissues.

From the point of view of chemical composition, natural resins are usually an important part of Chinese lacquers and consist mainly of neutral and acidic mixtures of monoterpenes, sesquiterpenes, diterpenes, and even triterpenes [39].

**Figure 4:**
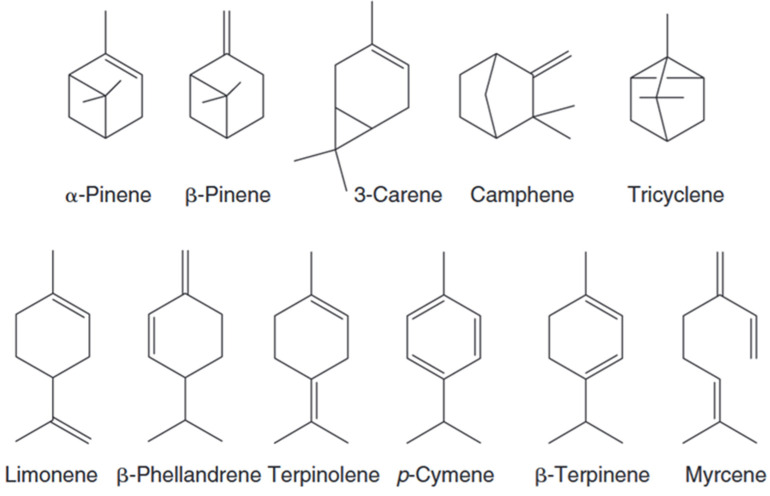
Chemical structure of monoterpenes.

The chemical structure of terpenes follows the “isoprene rule”, i.e., a chain or ring skeleton is formed by the head-to-tail connection of isoprene units. Natural terpenes often contain double bonds (e.g., the intracyclic double bond of α-pinene) or chiral centers (e.g., the cyclicity of (+)-α-pinene), and their stereo configuration directly affects their biological activity. Terpenes in lacquer are predominantly unoxidized hydrocarbons and are chemically stable, although they may undergo isomerization or cleavage reactions at high temperatures (Figure 4) [40].

Monoterpenes with strained alkenes (α-pinene, β-pinene, 3-carene) or conjugated systems (limonene, terpinolene) exhibit high reactivity toward atmospheric oxidation. Strained bicyclic bonds form skin-sensitizing epoxides (e.g., limonene oxide) and hydroperoxides (e.g., 3-carene hydroperoxide), while conjugated dienes generate electrophilic endoperoxides (e.g., ascaridole from terpinolene) and α,β-unsaturated ketones (e.g., carvone from limonene). Myrcene’s terminal alkene oxidizes to allergenic aldehydes. These electrophilic oxidation products act as haptens, binding dermal proteins to provoke allergic contact dermatitis.

Table 5 compares the physicochemical properties and safety of three terpenoids—limonene, pinene, and isoprene [41].

## 3. Detection Techniques

### 3.1. Static Analytical Techniques: Headspace–GCMS Coupling

Currently, for trace VOCs, static headspace [42], headspace solid-phase microextraction (SPME [43,44], purge trap [45,46,47], liquid–liquid extraction [48], and supercritical solid-phase microextraction (SSPME [49] are commonly used for extracting or concentrating the target substances, which are then quantitatively analyzed via GC or GC/MS. 

Headspace–gas chromatography–mass spectrometry (HS-GC/MS) is the current method for the detection of volatiles from large paints and is suitable for the qualitative and quantitative analysis of trace VOCs in a laboratory environment.

Headspace sampling seals the sample (e.g., raw lacquer) in a headspace vial to equilibrate the distribution of volatiles in the gas–liquid phase. The top gas is extracted through a gas-tight needle to avoid matrix interference. Gas chromatography (GC) separation means that the volatiles are carried by a carrier gas (e.g., helium) into a capillary column, and the efficient separation of compounds with different boiling points is achieved via programmed temperature increase. The separated components enter the mass spectrometry ion source, where they are electronically bombarded and fragmented into characteristic ions, which are characterized by matching the mass spectra to the NIST database.

Static headspace–gas chromatography–mass spectrometry (HS-GC/MS) is based on the principle of the equilibrium partitioning of volatile components between the sample and the gas phase, and allows the highly efficient analysis of volatile and semi-volatile compounds in solid and liquid samples [50,51].

Compared with traditional solvent extraction or solid-phase microextraction, static headspace–GC/MS does not require complex pre-treatment, can significantly reduce the contamination of non-volatile matrix components to the system, and is performed through automated injection to achieve high throughput and good reproducibility; at the same time, it is also the mainstream means for the quantitative analysis of VOCs [52]. Static headspace–GC/MS has been widely used in food aroma, biological sample, and environmental monitoring [53,54,55]. In recent years, it has also been gradually used in the study of large lacquer VOCs, allowing the real-time monitoring and quantitative evaluation of the release behavior of VOCs during lacquer processing by directly analyzing the main components such as α-pinene, limonene, and sesquiterpenes.

Headspace gas chromatography (GC) and mass spectrometry (MS) were used to analyze the major aromatic compounds in birch lacquer [19].

The GC conditions for the analysis of lacquer aroma were as follows. The injection and detector temperatures were 230 °C. The temperature of the thermostatic bath was maintained at 35 °C for 5 min and then ramped up to 200 °C at a rate of 2 °C/min. Helium was used as the carrier gas at a flow rate of 30 cm/s, and the chromatographic column used was a DB-Wax (60 m × 0.25 mm) column.

**Figure 5:**
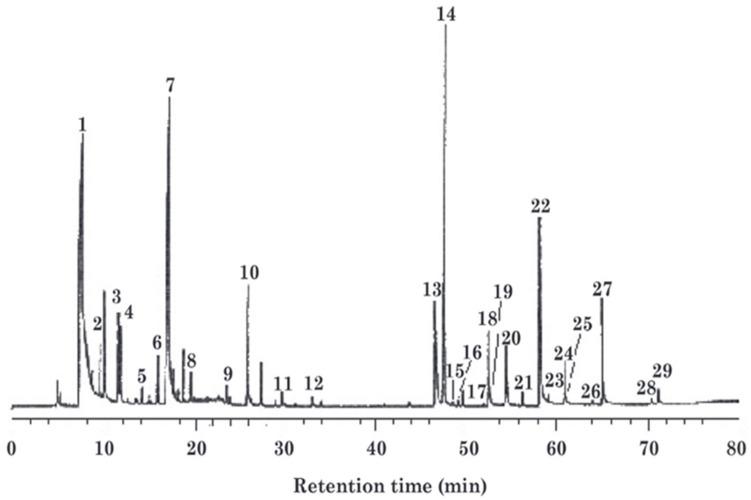
Mass spectra of volatile compounds in lacquer juice.

There are 29 real peaks in the total ion chromatogram (TIC) of the volatile compounds of lacquer sap [19]. The mass spectra are shown in Figure 5.

The early eluting peaks (1–14, 0–20 min) correspond to volatile monoterpenes (e.g., α-pinene, limonene, p-cymene). These contribute to respiratory irritation and acute toxicity; their abundance is reduced by distillation during lacquer processing. Mid-range peaks (15–24, 20–50 min) represent sesquiterpenes (e.g., caryophyllene, farnesene), which oxidize into skin-sensitizing epoxides and lactones (allergenicity risk). Late peaks (25–29, 50–80 min) are diterpenes or triterpenes (e.g., abietic acid, lupeol), linked to bioaccumulation and potential hepatotoxicity; thermal processing (e.g., heating) degrades these into irritants such as retene.

### 3.2. Photoionization Detector (PID)

A photoionization detector (PID) is a gas detection technology based on the principle of photoionization. A photoionization detector detects gases whose ionization potential is below the energy level of the radiation from an ultraviolet light source, and whose high-energy ultraviolet radiation can ionize most organic and some inorganic substances in the air while leaving the basic components of the air such as N_2_, O_2_, CO_2_, H_2_O unionized (the ionization potentials of these substances are greater than 11 eV) [56].

The core component is the vacuum ultraviolet (VUV) light source, usually krypton (Kr) or argon (Ar), which is used with other gases to generate specific energy photons, with a common photon energy of 10.6 eV or 11.7 eV. When the ionization energy of the gas molecules to be measured is lower than the energy of the VUV photons, the molecules absorb the photon energy and ionize, generating ions and electrons. In the ionization chamber, the ions and electrons move directionally under the action of the electric field, forming a weak electric current, which is processed by the amplifier circuit and converted into an electric signal proportional to the gas concentration, thereby allowing the quantitative detection of the gas concentration [57].

VOC monitoring using a PID is characterized by high monitoring accuracy, fast response timed, a long lifetime, and the ability to monitor VOCs non-destructively and at atmospheric pressure [58].

Although PIDs cannot completely distinguish isomers (e.g., o- and para-substituted derivatives of lacquer phenols), their significant advantages are in their sensitivity and real-time and on-site applicability, make them ideal for the rapid monitoring and risk assessment of lacquer volatiles. In practical application, they can be combined with meteorological chromatography (GC) and other separation techniques to further improve the component identification ability, forming a “rapid screening–accurate characterization” detection system and providing technical support for the detection of lacquer volatiles.

### 3.3. Fourier Transform Infrared Spectroscopy (FTIR)

Fourier transform infrared spectroscopy (FTIR) is a technique for the analysis and detection of compounds based on the absorption of infrared light by substances. The wavelength range of infrared light is roughly 0.78–1000 μm, corresponding to wave numbers 4000–10 cm⁻¹. When infrared light strikes a sample, the molecules of the sample selectively absorb infrared light at a specific frequency, which triggers a jump in the vibrational and rotational energy levels of the molecules.

Fourier transform infrared spectroscopy (FTIR) is a new detection technique that has emerged in recent years and is known as “molecular fingerprinting”. It does not require the destruction of cells or the addition of any chemical reagents, and only a small amount of biomass can be analyzed [59].

Due to the advantages of FTIR technology, which does not require pre-processing, has a fast response time, and allows simultaneous multi-component measurements, it has been widely used as an effective trace gas measurement method in various fields [60]. Since volatile organic compounds (VOCs) have significant infrared characteristic spectra [61], Fourier transform infrared spectroradiometers (FTIRs) are suitable for the detection of volatiles from large lacquers due to their high sensitivity and resolution, as well as the possibility of real-time simultaneous multi-component telemetry.

However, there are some challenges in the use of FTIR technology in the detection of lacquer volatiles, such as the complex composition of lacquer volatiles, and there may be some overlapping absorption peaks, which require more complex spectral analysis methods. For some volatiles at low concentrations, it may be necessary to combine FTIR spectroscopy with other techniques for enrichment and detection.

### 3.4. Comparative Evaluation of Analytical Methods

A systematic comparison of key VOC detection techniques is essential for selecting appropriate methods in lacquer research. Table 6 summarizes the performance metrics of mainstream technologies.

## 4. Mechanisms

### 4.1. Unit Phenolics

Unitary phenolics have strong redox properties and are capable of generating free radicals in the body, thereby inducing cellular damage and inflammatory responses [62]. Studies have shown that upon contact with the human body, unitary phenolics can bind to proteins on the surface of the skin or respiratory tract to form semi-antigens, which activate T-helper cells (Th2) in the immune system, leading to allergic reactions [63]. Studies have shown that unit phenols are capable of releasing reactive oxygen species (ROS) such as superoxide anions, hydrogen peroxide, and hydroxyl radicals during oxidation, and these reactive molecules can cause irreversible damage to biofilm lipids, proteins, and DNA [64].

In addition, the metabolic processes of unitary phenolics in the body may also exacerbate the production of free radicals. When these compounds enter the body, they are bio-transformed by the cytochrome P450 enzyme system in the liver, and this process is often accompanied by the generation of large amounts of free radicals [65].

Once these free radicals exceed the scavenging capacity of the body’s antioxidant system, they lead to a state of oxidative stress. Oxidative stress is an imbalance in the redox system caused by the production of excess free radicals or exceeding the scavenging capacity of the organism when it is exposed to external stimuli [66]. Previous studies have found that oxidative stress can cause oxidative damage to tissue cells and is the basis for the development of many metabolic diseases and cancers [67,68].

Thus, the presence of unitary phenols in lacquer volatiles may significantly increase an individual’s susceptibility to non-contact allergy through a free radical generation mechanism.

### 4.2. Aldehydes and Ketones

Aldehyde and ketone volatiles (e.g., acrolein, formaldehyde) in large lacquers have extremely low boiling points (52.7 °C for acrolein, −19 °C for formaldehyde), and volatilize easily into a gaseous state at room temperature, with a pungent odor (perception thresholds as low as 0.1 ppm). Their vapor density is greater than that of air, so they easily accumulate in the working environment; they are both water-soluble and fat-soluble, so they can penetrate the skin and mucous membranes of the respiratory tract, thereby exacerbating the risk of biological exposure.

Most aldehydes and ketones irritate skin and mucous membranes and poison the central nervous system [69]. Most of these compounds are irritating and toxic, causing strong irritation to the eyes, nose, skin, lungs and respiratory tract, and they are carcinogenic, teratogenic, and mutagenic [70,71,72].

Aldehydes and ketones are also widely found in lacquer. Taking vanillin as an example, its molecular structure contains functional groups such as aldehyde group and methoxy group. The aldehyde group has high electrophilicity and can react with nucleophilic groups of biological macromolecules such as proteins and nucleic acids. When vanillin comes into contact with the skin or respiratory mucosa, its aldehyde group may condense with the amino groups of cell surface proteins to form adducts such as Schiff bases. In addition, acetone may also have a certain impact on the skin’s barrier function, making it easier for the skin to absorb other allergens.

### 4.3. Terpenes

Terpenes in lacquer belong to a large group of natural hydrocarbon compounds whose basic skeleton consists of isoprene units (C_5_H_8_).

The so-called terpene sensitivity is due to oxidation products. In patch tests of pure and air-exposed turpentine or isolated grades in dermatitis patients, it was observed that the “eczematous” component of turpentine was apparently not related to the pure hydrocarbon terpenes C (10) H16 but rather to the products formed by their oxidation. It has also been shown that α-pinene, β-pinene, and limonene are auto-oxidized, although Δ3-pinene is oxidized at a much faster rate [73].

Many articles have clearly shown that terpenes can react with air to form oxidation products [74,75,76], and that these oxidation products (mainly hydroperoxides) are very potent skin allergens [77].

Terpene hydroperoxides have been identified as strong sensitizers [78]. The human sensitizing potential and potency of several hydroperoxides have been determined in vitro in human cells, showing comparable results to in vivo tests [79]. Intensive studies have shown that terpene hydroperoxides form reactive radical intermediates capable of modifying amino acids in epidermal proteins [79,80,81].

In the case of β-stigmastene, β-stigmastene oxidation is a major skin irritant, and the skin is easily sensitized by β-stigmastene. After 10 weeks of exposure to the air, its irritation is weakened. Furthermore, β-stilbene oxide is a medium-strength allergen and studies have shown that β-stilbene is a sensitizer [82]. The reason why β-stilbene is a potent allergen is because β-stilbene’s double bond is easily oxidized [83]. The reason why the double bond of β-stilbene is easily oxidized is because β-stilbene is formed when it is exposed in the air to peroxide, which is very unstable in nature, converts to a primary peroxide product, and then quickly converts to a more stable oxidation product, a moderately sensitized oxide [84].

Specifically, terpenoids are easily oxidized in air to produce a series of secondary products such as hydroperoxides and epoxides. These secondary products not only have high chemical reactivity but also bind non-specifically to proteins or other biomolecules in the skin, thereby forming new antigenic epitopes and triggering an immune response [85].

### 4.4. Toxicological Relevance of Major VOC Classes in Lacquer Processing

Based on the above introduction, the allergy mechanisms of several major categories of volatile organic compounds in the lacquer were compared in Table 7.

## 5. Prevention

### 5.1. Prevention and Control Strategies Based on Detection Technologies

Headspace–gas chromatography–mass spectrometry (HS-GC/MS) as a standard detection method plays an important role in the qualitative and quantitative analysis of trace VOCs in laboratory settings. In actual production, the technique can be used regularly to detect large paint volatiles in the workplace to keep abreast of changes in the composition and content of the volatiles.

In situ mass spectrometry real-time tracking technology can be used to monitor the dynamic release process of VOCs online and continuously. Monitoring equipment based on this technology can be installed in large lacquer processing workshops to grasp the pattern of volatile release in real time. Once an instantaneous increase in the concentration of sensitized volatiles is detected, ventilation equipment is immediately activated to reduce the concentration of volatiles in the workshop.

### 5.2. Prevention and Control Strategies Based on Sensitization Mechanisms

#### 5.2.1. For Unit Phenols

Monophenols induce allergic reactions by generating free radicals due to their redox properties. Antioxidant protective equipment can be developed, such as protective gloves and masks containing antioxidant components. Vitamin C, vitamin E, and other antioxidants can be added to the protective materials, so that when unitary phenolic substances come into contact with the protective equipment, the antioxidants can inhibit the generation of free radicals and reduce the risk of allergy. Some studies have shown that vitamin C can effectively scavenge reactive oxygen species such as superoxide anion radicals and block free radical chain reactions [86].

In lacquer processing, the oxidation of unit phenolics can be inhibited by adding antioxidants. For example, by adding an appropriate amount of di-tert-butyl-p-cresol (BHT) [87] to the lacquer, it can preferentially react with oxygen, preventing the oxidation of unit phenols to generate free radicals and reducing the risk of allergy from the source.

#### 5.2.2. For Aldehydes and Ketones

Aldehyde and ketone volatiles have low boiling points, accumulate easily, and are irritating and toxic. In the place of storage and use of lacquer, install a high-efficiency ventilation system to ensure air circulation. The ventilation system should be reasonably designed according to the area of the workshop and the amount of lacquer used, such as 10–15 times per hour, to discharge the volatile aldehydes and ketones in time and reduce the concentration in the workshop. As lacquer is both water-soluble and fat-soluble, an air purification device can be set up to purify the air via water curtain spraying combined with activated carbon adsorption. Water curtain spraying removes some of the water-soluble aldehydes and ketones, and activated carbon adsorption further removes the remaining volatiles, reducing the risk of worker exposure.

#### 5.2.3. For Terpenes

Terpenes are easy to oxidize to generate sensitizing products. Adding antioxidants to the lacquer to inhibit the oxidation reaction, such as adding propyl gallate [88] and other antioxidants, can effectively delay the oxidation of terpenes and reduce the generation of sensitized oxidation products. In the workshop environment, control the air humidity and temperature to avoid a high-temperature and high-humidity environment to accelerate terpene oxidation. For example, the temperature of the workshop should be controlled at 20–25 °C and the relative humidity at 40–60% to reduce the rate of terpene oxidation.

Alternatively, the use of fungal fermentation, such as black-shanked charcoal hornbeam [89], produces cytochrome P450 enzymes that allow terpenes to oxidize prematurely, thereby depriving them of their sensitizing properties and achieving protection.

### 5.3. Cyclodextrin and Chitosan Embedding Technology

Cyclodextrins (CDs) are a class of biocompatible cyclic oligosaccharides with hydrophobic inner cavities and hydrophilic outer surfaces, which are often used to construct supramolecular systems due to their inherent homogeneous cavities and the presence of a large number of -OH groups [90]. Chitosan, a polysaccharide found in the shells of many crustaceans, has emerged as a reliable material for the preparation of nanoparticles, as it is a biodegradable, biocompatible, non-toxic, and inexpensive biopolymer [91]. Using the cyclodextrin and chitosan encapsulation technique, sensitized substances such as lacquer phenols in lacquer can be effectively treated.

Lacquer phenol is a strong sensitizer, and its molecular structure is capable of interacting with certain components of the body’s immune system to trigger an allergic reaction. When cyclodextrins and chitosan are used to encapsulate lacquer phenols, the cyclodextrins bind to the lacquer phenol molecules through their hydrophobic inner cavities, encapsulating the lacquer phenols. Chitosan surrounds the cyclodextrin to form a protective barrier. This spatial site-blocking effect results in a significant reduction in the antigenicity of the enantiomers, thereby reducing the possibility of their triggering an allergic reaction. In practice, a solution containing the cyclodextrin–chitosan embedding system can be mixed with the lacquer, and the embedding process can be fully carried out by appropriate stirring and reaction conditions. The amount of sensitized lacquer phenols released during use of the lacquer is significantly reduced, reducing the risk of allergic reactions to the user.

### 5.4. Laccase Active Site-Directed Modification

Laccase plays a key role in the drying and curing process of lacquer by catalyzing the oxidative polymerization of substances such as lacquer phenols. However, the toxic products such as quinones catalytically produced by laccase in this process may trigger allergic reactions. Targeted modification of the laccase active site through genetic engineering strategies can effectively inhibit the production of quinone toxic products.

In the protein structure of laccase, there are specific active sites that determine its catalytic activity and reaction specificity. Using modern genetic engineering techniques, such as targeted mutagenesis [92], the gene sequences encoding the active sites of laccase can be precisely modified. By changing the amino acid residue composition of the active site, the catalytic activity and selectivity of laccase can be adjusted.

### 5.5. Nanofiber Membrane High-Efficiency Interception Technology

Electrostatic spinning technology is one of the important methods for the preparation of nanofibers. due to its simple process and the preparation of nanofibers with the advantages of high porosity levels, small diameters, and light weights, it has received extensive attention and research, and at present, electrostatic spinning fibers are mainly used in the field of biological tissue engineering and for adsorption and filtration of air and water [93]. It has significant advantages in the interception of VOCs in large paint volatiles.

During electrostatic spinning, the polymer solution jet overcomes the polymer surface tension under a high-voltage electrostatic field, is stretched by electrostatic force, and solidifies (as the solvent evaporates or the melt cools down) into microfibers. The molecules of VOCs in the volatiles of large paints undergo adsorption–filtration phenomenon when they diffuse in the air and come into contact with the nanofiber membrane. On the one hand, the high specific surface area of the nanofiber membrane gives it a strong adsorption capacity, and the VOC molecules will be adsorbed on the fiber surface.

In practical applications, nanofiber membranes can be made into PPE such as protective masks and suits. When workers wear such equipment while they are in contact with the lacquer, the nanofiber film can efficiently intercept the VOCs volatilized by the lacquer and reduce the chances of the sensitized substances entering the human respiratory system and contacting the skin, thereby playing a good protective role.

### 5.6. Policies and Regulations

The current domestic and international regulations for VOCs in the lacquer industry include China’s GB 18581-2020 (≤550 g/L for solvent-based and ≤250 g/L for water-based lacquers) [94], the EU REACH restrictions on formaldehyde, and the US EPA VOC emission rules. Policy efforts have led to improved safety via strict standards and certifications. Future steps should integrate real-time monitoring, promote bio-based materials, enhance worker health screenings, and harmonize global testing protocols.

### 5.7. Comparison of Preventive Strategies

A concise summary of the proposed mitigation strategies, the experimental rationale supporting them, the identified limitations, and the critical challenges associated with their industrial implementation was listed in Table 8.

## 6. Summary

The primary component of Chinese lacquer is urushiol (60–65%), a catechol derivative with unsaturated aliphatic side chains that confers both the material’s superior performance and potent allergy. Volatile emissions predominantly include aldehydes, ketones, terpenes, and trace monomeric phenols. Low-boiling-point aldehydes and ketones (e.g., acrolein, formaldehyde) exhibit high volatility and irritancy, triggering inflammatory responses via respiratory or dermal exposure. Terpenes (e.g., α-pinene) generate secondary allergens such as hydroperoxides through oxidation, contributing to non-contact allergy.

Static headspace–GC/MS remains the benchmark analytical method for detecting trace VOCs under controlled laboratory conditions, combining gas–liquid equilibrium sampling with high-resolution mass spectrometry. Complementary techniques such as photoionization detection (PID) and Fourier transform infrared (FTIR) spectroscopy demonstrate growing applicability for lacquer volatile analyses.

Allergy mechanisms involve multiple pathways: monomeric phenols oxidize to form free radicals, inducing oxidative stress and protein adducts that activate Th2 immune responses; aldehydes and ketones covalently bind to skin proteins via aldehyde groups, altering the antigenicity while their lipophilicity elevates bioaccumulation risks; oxidized terpenes modify epidermal components to create neo-antigenic epitopes, synergistically amplifying urushiol’s sensitized effects.

The mitigation strategies adopt a multi-tiered approach. The source control employs antioxidant additives (e.g., butylated hydroxytoluene, ascorbic acid) to suppress urushiol oxidation and radical formation, alongside cyclodextrin and chitosan encapsulation to reduce bioactive urushiol exposure. The process enhancements include the enzymatic modification of laccase active sites to minimize toxic quinone byproducts and fungal pretreatment to degrade terpene precursors. Engineering controls can be used to integrate nanofiber-based personal protective equipment for particulate filtration and ventilation systems for real-time VOC reductions. Health management protocols can be used to establish allergy screening and tiered protection standards to improve occupational safety awareness.

Future research studies should focus on three key areas: first, deciphering how complex mixtures of volatile organic compounds (VOCs) in lacquer, such as urushiol, terpene oxides, and aldehydes, enhance allergy through synergistic interactions, as well as the mechanisms behind individual variations in allergic responses, including the role of genetic differences (e.g., immune-related gene polymorphisms); second, developing portable detection tools, such as AI-powered sensors integrated with machine learning for real-time VOC fingerprinting, graphene-based nanobiosensors for ultra-sensitive detection (down to sub-ppb levels), and mobile Fourier transform infrared (FTIR) spectrometers for on-site rapid screening, to improve the real-time monitoring of VOCs in workshop environments; third, driving technological innovation through interdisciplinary collaboration, such as using gene editing techniques to cultivate low-allergy lacquer tree varieties, designing nanofiber protective membranes with antioxidant functional groups to efficiently adsorb volatile allergens, and establishing lifecycle environmental risk assessment models for lacquer craftsmanship via industrial ecology approaches to achieve green upgrades of traditional lacquer art.

## Figures and Tables

**Table 1:** Differences in the side-chain structures of three lacquer trees.

Component	Source Tree Species	Biological Relevance of Side Chain
Urushiol	*Rhus vernicifera*	High allergenicity: Unsaturated chains readily oxidize to quinones, forming potent skin-sensitizing haptens
Laccol	*Rhus succedanea*	Reduced sensitization: Saturated chains resist oxidation, decreasing hapten formation and allergic responses
Thitsiol	*Melanorrhoea usitata*	Dual reactivity: Aromatic groups enable radical-based polymerization while aliphatic chains modulate tissue penetration

**Table 2:** Results of GC analyses of volatile compounds in lacquer sap of Lacustrum officinale [32].

Number	Retention Time (min)	Peak Area (%)	Chemical Composition
1	7.1	49.3	Ethyl formate
2	9.4	1.5	ethyl acetate
3	11.6	6.5	2-Propanol
4	11.8	4.8	ethanol
5	14.0	0.3	MHC (monoterpene hydrocarbons)
6	15.8	0.7	MHC (monoterpene hydrocarbons)
7	16.8	31.4	1-Propanol
8	19.4	0.5	MHC (monoterpene hydrocarbons)
9	23.7	0.1	MHC (monoterpene hydrocarbons)
10	25.9	4.3	butanol
11	29.9	0.4	MHC (monoterpene hydrocarbons)
12	33.0	0.2	limonene
13	46.8	7.0	acetic acid (CH3COOH)
14	47.7	30.4	α-Longifolene
15	48.2	0.5	alpha-Elangene
16	48.4	0.5	alpha-Cobalene
17	48.9	0.9	SHC (Sesquiterpene Hydrocarbon)
18	53.0	6.3	propanoic acid
19	53.1	2.9	alpha-Cedrene
20	54.9	4.0	isobutyric acid
21	56.3	1.6	α-Bergamotene
22	58.4	3.4	butyric acid
23	59.1	0.8	alpha-Himalayan alkene
24	61.2	3.4	isovaleric acid
25	61.3	3.0	alpha-Serpentene
26	64.1	0.9	β-Celestene
27	65.6	11.7	valeric acid
28	70.4	0.3	δ-Cadinene
29	71.2	3.0	hexanoic acid

**Table 3:** Breakdown of aldehydes and ketones.

Aldehydes	Ketones	Aromatic Aldehydes
Formaldehyde, Acetaldehyde, Propionaldehyde, Butyraldehyde, Pentanal, Hexanal, Acrolein, Crotonaldehyde	Acetone Butanone Cyclohexanone	Benzaldehyde m-Tolualdehyde p-Tolualdehyde 2,5-Dimethylbenzaldehyde

**Table 4:** Physical properties of aldehydes and ketones.

Characterization	Aldehyde	Ketone
Boiling point range	19.5 °C~179 °C	56.05 °C~155.6 °C
volatility	Very strong	Medium
Density range (g/cm^3^)	0.788 (acetaldehyde)~1.044 (benzaldehyde)	0.784 (acetone)~0.948 (cyclohexanone)
Solubility (water)	Small molecules are soluble Macromolecule insoluble	small molecules miscible Low solubility of macromolecules
Odour characteristics	Irritating or peculiar odour	Mild or distinctive odour
Melting point range	−123.5 °C~−26 °C	−94.7 °C~−45 °C
environmental impacts	high risk of respiratory exposure	High density substances

**Table 5:** Properties of limonene, pinene, and isoprene.

Causality	Limonene	Pinene	Isoprene
Chemical name	p-Mentha-1,8-diene	-	2-Methyl-1,3-butadiene
Synonyms	4-Isopropenyl-1-methylcyclohexene	-	Beta-methylbivinyl,2-methylbutadiene
Enantiomer (chemistry)	D-Limonene (R-type) L-Limonene (S-type)	α-Pinene β-Pinene	
Racemate	DL-Limonene (Dipentene)	-	-
Chemical formula	C_10_H_16_	C_10_H_16_	C_5_H_8_
Molecular mass	136.24 g/mol	136.24 g/mol	68.11 g/mol
Appearances	Transparent colourless liquid	Transparent colourless liquid	Transparent colourless liquid
Melting point	−95.2 °C	−64 °C	−145.95 °C
Boiling	176 °C	155 °C	34.067 °C
Density (20° C)	0.8411 g/cm^3^	0.858 g/cm^3^	0.681 g/cm^3^
SMILES ^(1)^	CC_1_=CCC(CC_1_)(C)C=C	CC_1_(C)C_2_CC_1_C(C)=CC_2_	C=C(C)C=C
CAS No. ^(2)^	138-86-3	80-56-8	78-79-5
IARC Class ^(3)^	3	3	2B

^(1)^ Simplified molecular input line entry system. ^(2)^ Chemical abstracts service registry numbers. ^(3)^ International Agency for Research on Cancer, Class 3; not classifiable as to its carcinogenicity to humans, Class 2B; possibly carcinogenic to humans.

**Table 6:** Comparative evaluation of VOC detection techniques.

Technique	Sensitivity	Throughput	Cost	Key Applications
HS-GC/MS	0.1–10 ppb	Low–Medium	High	Lab-based speciation, quantification
PID	0.1–100 ppm	High	Low–Medium	Real-time field screening
FTIR	1–100 ppm	Medium	Medium–High	Multi-component monitoring

**Table 7:** Toxicological relevance of major VOC classes in lacquer processing.

Class	Typical Concentration Ranges (Lacquer Processing)	Major Allergy Mechanisms
Phenols	0.01–0.5 mg/m^3^ (monomeric phenols); skin contact: 10–100 μg/cm^2^ (urushiol)	Oxidation to ortho-quinones (haptens) → protein conjugation → Type IV hypersensitivity (Th2 response).
Aldehydes	Formaldehyde: 0.05–5 ppm; Acrolein: 0.1–5 mg/m^3^	Protein crosslinking → antigenic epitopes → T-cell-mediated allergic activation.
Ketones	Acetone: 50–200 mg/m^3^; Cyclohexanone: 5–20 mg/m^3^	Skin barrier disruption → enhanced allergen penetration; epigenetic inflammation promotion.
Terpenes	α-Pinene: 10–500 mg/m^3^; Limonene: 5–100 mg/m^3^	Oxidation products (hydroperoxides) → NLRP3 inflammasome activation → Th2-skewed immunity

**Table 8:** Strategy evaluation.

Mitigation Strategy	Key Experimental Support	Limitations	Industrial Challenges
Cyclodextrin/Chitosan Embedding	Encapsulates urushiol via hydrophobic cavities and spatial barriers.	No quantitative efficiency data; potential impact on film properties.	Compatibility with traditional recipes; process optimization needed.
Laccase Modification	Targeted mutations reduce quinone byproducts through altered catalytic pathways.	Complex genetic engineering and microbial expression requirements.	High production costs; acceptance in traditional craftsmanship.
Antioxidant Protective Equipment	Antioxidant additives (e.g., vitamin C) inhibit free radical formation.	Short-lived efficacy; limited protection against airborne VOCs.	Disposable equipment costs; worker compliance with PPE.
Terpene Oxidation Control	Antioxidants (e.g., propyl gallate) delay oxidation; controlled humidity and temperature reduces reaction rates.	Requires energy-intensive environmental control; potential storage stability issues.	Retrofitting production lines; additive–resin compatibility.
Nanofiber Membrane Filtration	High-surface-area membranes adsorb and filter VOCs for PPE and ventilation.	Clogging risk with high VOC loads; balance between breathability and efficiency.	Scalable manufacturing costs; long-term material durability.

## Data Availability

Data are contained within the article.
